# Editorial: Insights in microbial physiology and metabolism: 2021

**DOI:** 10.3389/fmicb.2022.1054708

**Published:** 2022-10-26

**Authors:** Biswarup Mukhopadhyay, Ulrike Kappler, Katarzyna Potrykus, Xinqing Zhao, Cornelia Welte

**Affiliations:** ^1^Department of Biochemistry, Virginia Tech, Blacksburg, VA, United States; ^2^School of Chemistry and Molecular Biosciences, The University of Queensland, Brisbane, QLD, Australia; ^3^Department of Bacterial Molecular Genetics, University of Gdansk, Gdansk, Poland; ^4^School of Life Sciences, Shanghai Jiao Tong University, Shanghai, China; ^5^Department of Microbiology, Radboud University, Nijmegen, Netherlands

**Keywords:** microorganisms, microbial metabolism, microbial physiology, enzymes, host—bacteria interaction, biotechnology

Metabolic diversity and adaptability drive the ability of microorganisms to colonize virtually all habitats on earth and to cope with extreme temperatures, pH and highly diverse nutrient profiles. Microbial metabolism is also emerging as a critical determinant of interactions between host organisms and the microorganisms associated with them and is also a resource as well as a tool in synthetic biology and biotechnology.

Despite decades of research, however, there is significant “metabolic dark matter” to be discovered in microorganisms, and this Research Topic highlights the many recent achievements in expanding our knowledge of microbial metabolism and discovering previously unknown, novel metabolic functions.

Several contributions to this Research Topic explore fundamental aspects of microbial physiology. Maintenance of cellular protein homeostasis is an essential process for all living cells. Matavacas and von Wachenfeldt have compiled the latest insights into this essential cellular function in the model bacterium *Bacillus subtilis* and also explore how protein homeostasis contributes to the survival of Gram-positive pathogens. Equally fundamental is the correct partitioning of DNA during cell division, where Mishra and Srinivasan have reviewed the latest insights into ParA ATPases.

Many bacterial cells contain alpha or beta carboxysomes, assemblies that contain Rubisco, carbonic anhydrase, and shell proteins and contribute to CO_2_ fixation *via* Rubisco. USF Genomics Class 2020 have provided a review of “atypical carboxysomes” that lack one or more of these conserved carboxysome components. The evidence suggests that atypical carboxysomes give rise to functional assemblies and most likely have altered functions that depend on the elements present.

Stress responses are essential for the ability of bacteria to survive in adverse environments, and responses to nutrient starvation are central to bacterial survival. Hagberg et al. have investigated how *Sinorhizobium meliloti* responds to phosphate and nitrogen stress while also aiming to understand the overlap of these two starvation responses. Interestingly, their work revealed only minimal overlap and concluded that phosphate stress is a more significant metabolic burden and takes precedence over nitrogen stress.

Stress responses were also a topic in papers that focussed on the physiology and metabolism of bacterial pathogens. Edelmann et al. have analyzed available data on the TisB Toxin/ Antitoxin system that is thought to induce the formation of “persister cells” that are resistant to, for example, antibiotic-induced stress. The authors conclude that the expression pattern for TisB is more consistent with a different role, namely maintenance of the persister state once other cellular stress responses have induced it.

In an investigation of the role of metabolism in pathogenesis, Yue et al. found that in the human pathogen *Francisella tularensis*, arginine and polyamine metabolism positively correlate with hypervirulence, while less virulent strains frequently lacked relevant genes.

The largest groups of papers focused on metabolic and physiological processes relevant to biotechnology or organisms used in biotechnological applications.

Du et al. reveal a previously unrecognized function of the known carbon metabolism regulator Cat8 from budding yeast *Saccharomyces cerevisiae* in controlling nitrogen utilization. These results provide novel insights in regulation of yeast nutrient metabolism networks. Focussing on a specific group of enzymes, Bhayani et al. provide new insights into the catalytic properties of ADP-glucose pyrophosphorylases from various bacterial species and their reactivity toward glucosamine, which is relevant to future applications.

Commercial applications are often limited by the efficiency of relevant enzyme systems or a lack of access to substrates. A review by Henrion et al. examines the applicability of control theory in a broader context, namely to population-wide gene expression and phenotypic changes. Tao et al. analyzed the role of X2 modules in the cellulosomal scaffolding protein in *Clostridium cellulolyticum* where X2 modules play a role in elevating cellulose degradation efficiency by stimulating the binding between the bacterial cells and cellulose. This provides new perspectives for microbial engineering to improve degradation of cellulose.

Gendron and Allen reviewed current knowledge of archaeal methyl-/alkyl-coenzyme M reductases and provided insights into how heterologous expression of these enzymes could enhance biotechnological applications in methane and alkane production.

Lastly, the removal of CO or CO_2_ from the atmosphere and their conversion into value-added products *via* the Wood-Ljungdahl pathway was reviewed by Lee et al.. The authors explore strategies to overcome growth limitations of acetogenic bacteria by engineering the Wood-Ljungdahl pathway, thereby increasing their potential as catalysts of this valuable transformation.

This collection of articles is an excellent example of the breadth and depth of research into microbial physiology and metabolism and its relevance to issues that range from fundamental research to applications in the health sciences and biotechnology industry. The research progress in this Research Topic provides novel insights in the field of microbial metabolism and metabolic engineering.

## Author contributions

UK drafted the editorial text. All authors contributed to the editing of the draft and generation of the final editorial. All authors contributed to the article and approved the submitted version.

## Conflict of interest

The authors declare that the research was conducted in the absence of any commercial or financial relationships that could be construed as a potential conflict of interest.

## Publisher's note

All claims expressed in this article are solely those of the authors and do not necessarily represent those of their affiliated organizations, or those of the publisher, the editors and the reviewers. Any product that may be evaluated in this article, or claim that may be made by its manufacturer, is not guaranteed or endorsed by the publisher.

